# Supplementary material for: Indicator of Inflammation and NETosis—Low-Density Granulocytes as a Biomarker of Autoimmune Hepatitis
Source: J Clin Med. 2022 Apr 13;11(8):2174. doi: 10.3390/jcm11082174 (PMC9026397; doi:10.3390/jcm11082174)
Supplement: Supplementary file 1 [file jcm-11-02174-s001.zip › jcm-1625420-supplementary.pdf]

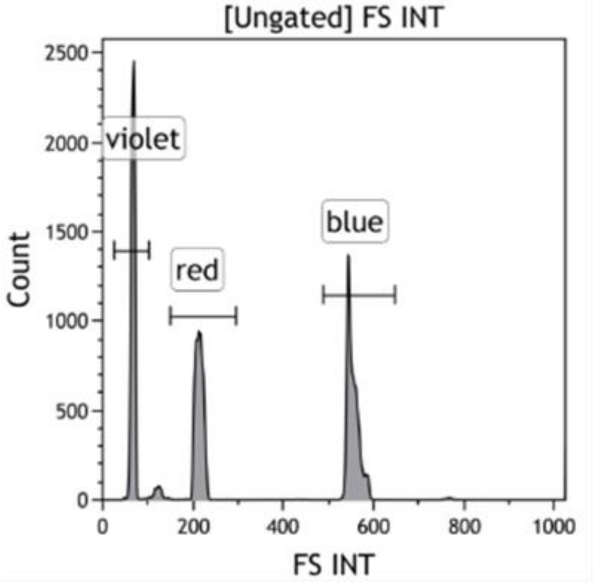

Supplementary Figure S1. Calibration of the violet (405 nm), red (633 nm) and blue (488 nm) laser signal (my own source).

Supplementary Table S1. Comparison of selected classical markers of inflammation and the percentage of LDG, including the fraction showing MPO expression in the control and study groups.

**Variable**	**Control** *n* = 20 **Median (Range)**	**AIH** *n* = 25 **Median (Range)**	*p*
WBC [10^3/uL]	5.8 (4.1–8.4)	6.2 (1.8–12.9)	0.1534
CRP [mg/L]	1.5 (1.0–8.9)	3.0 (0.9–13.9)	0.0157 *
LDG [%]	0.1 (0.0–1.7)	1.2 (0.2–24.5)	0.0001 *
LDG MPO+[%]	0.3 (0.0–2.5)	0.8 (0.1–67.4)	0.0017 *

AIH—Autoimmune hepatitis, CD—Cluster of differentiation, CRP—C-reactive protein LDG—Low Density Granulocytes, MPO—Myeloperoxidase, WBC—White blood cells. *—Statistically significant result.

Supplementary Table S2. Comparison of selected biochemical and inflammatory markers, including the percentage of LDG and the LDG fraction showing MPO expression in the group of patients with LC or non-LC in the course of AIH.

Variable	LC<br>*n* = 8<br>Median<br>(Range)	Non-LC<br>*n* = 17<br>Median<br>(Range)	*p*
AST [IU/L]	55.0<br>(25.0–204.0)	46.0<br>(14.0–2001.0)	0.5202
ALT [IU/L]	56,5<br>(17.0–101.0)	68.0<br>(20.0–1445.0)	0.9268
GGTP [IU/L]	74.0<br>(7.0–595.0)	117.0<br>(11.0–952.0)	0.5607
GPR	1.9<br>(0.1–24.9)	1.2<br>(0.1–6.9)	0.0085 *
AAR	1.2<br>(0.7–2.0)	0.8<br>(0.3–1.5)	0.0708
FIB-4	3.6<br>(1.6–17.4)	1.3<br>(0.6–14.9)	0.0044 *
APRI	2.0<br>(0.5–10.4)	0.6<br>(0.2–29.1)	0.0346 *
WBC [10^3/μL]	4.6<br>(1.8–6.2)	7.2<br>(4.6–12.9)	0.0013
PLT [10^3/μL]	85.0<br>(47.0–201.0)	246.0<br>(89.0–404.0)	0.0402
CRP [mg/L]	3.2<br>(1.0–7.3)	3.0<br>(0.9–13.9)	1.0000
LDG	0.4<br>(0.2–17.1)	2.1<br>(0.3–24.5)	0.0577
LDG MPO+	0.5<br>(0.3–8.1)	1.4<br>(0.1–67.4)	0.3425

AAR—(Aspartate aminotransferase—to-Alanine aminotransferase Ratio), APRI—Aspartate aminotransferase-to-Platelet Ratio Index, ALT—Alanine Aminotransferase, AST—Aspartate Aminotransferase, CD—Cluster of differentiation, CRP—C-Reactive Protein, GGTP—Gamma Glutamyl Transpeptidase, FIB-4—Fibrosis-4, GPR—Gamma-glutamyl-transpeptidase-to-Platelet Ratio, LC—Cirrhosis, LDG—Low Density Granulocytes, MPO—Myeloperoxidase, PLT—Platelet, WBC—White Blood Cells. *—Statistically significant result.
